# Characteristics and Clinical Course of Adult Inpatients with SARS-CoV-2 Pneumonia at High Altitude

**DOI:** 10.1155/2021/5590879

**Published:** 2021-06-08

**Authors:** Javier Leonardo Galindo, Juan Ricardo Lutz, María Alejandra Izquierdo, Katherine Parra, Lina María Prieto, Jorge Alberto Carrillo

**Affiliations:** ^1^Department of Pneumology, Hospital Universitario Mayor Méderi, Bogotá, Colombia; ^2^School of Medicine, Universidad Del Rosario, Bogotá, Colombia; ^3^Department of Surveillance and Epidemiology, Hospital Universitario Mayor Méderi, Bogotá, Colombia; ^4^Department of Radiology, Hospital Universitario Mayor Méderi, Bogotá, Colombia

## Abstract

**Background:**

SARS-CoV-2 has spread worldwide with different dynamics in each region. We aimed to describe the clinical characteristics and to explore risk factors of death, critical care admission, and use of invasive mechanical ventilation in hospitalized patients with SARS-CoV-2 pneumonia in a high-altitude population living in Bogotá, Colombia.

**Methods:**

We conducted a concurrent cohort study of adult patients with laboratory-confirmed SARS-CoV-2 pneumonia. Demographic, clinical, and treatment data were extracted from electronic records. Univariate and multivariable methods were performed to investigate the relationship between each variable and outcomes at 28 days of follow-up.

**Results:**

377 adults (56.8% male) were included in the study, of whom 85 (22.6%) died. Nonsurvivors were older on average than survivors (mean age, 56.7 years [SD 15.8] vs. 70.1 years [SD 13.9]; *p* ≤ 0.001) and more likely male (28 [32.9%] vs. 57 [67.1%]; *p*=0.029). Most patients had at least one underlying disease (333 [88.3%]), including arterial hypertension (149 [39.5%]), overweight (145 [38.5%]), obesity (114 [30.2%]), and diabetes mellitus (82 [21.8%]). Frequency of critical care admission (158 [41.9%]) and invasive mechanical ventilation (123 [32.6%]) was high. Age over 65 years (OR 9.26, 95% CI 3.29–26.01; *p* ≤ 0.001), ICU admission (OR 12.37, 95% CI 6.08–25.18; *p* ≤ 0.001), and arterial pH higher than 7.47 (OR 0.25, 95% CI 0.08–0.74; *p*=0.01) were independently associated with in-hospital mortality.

**Conclusions:**

In this study of in-hospital patients with SARS-CoV-2 pneumonia living at high altitude, frequency of death was similar to what has been reported. ICU admission and use of invasive mechanical ventilation were high. Risk factors as older age, ICU admission, and arterial pH were associated with mortality.

## 1. Introduction

In December 2019, a cluster of cases of severe pneumonia of unknown cause were identified in Wuhan, China. A novel strain of betacoronavirus called severe acute respiratory syndrome coronavirus 2 (SARS-CoV-2) was identified as the etiologic agent, and coronavirus disease 2019 (COVID-19) the disease it causes [1]. Since then, SARS-CoV-2 has spread worldwide and the number of cases and deaths have followed an exponential trend [2]. As of April 30th, 2021, more than 2.8 million cases and more than 70 thousand deaths of COVID-19 have been reported in Colombia [3].

Factors such as male sex, increasing age, diabetes, cardiovascular diseases, chronic respiratory diseases, and obesity have been associated with increased risk of death by COVID-19 [4]. Nowadays Latin America is a hotspot for the pandemic; however, there is still a lack of information about the clinical features and prognostic factors of this disease in this region [2].

Latin America and Colombia have singularities that could influence clinical presentation of COVID-19 due to their healthcare systems, their social composition, and the diversity of their geography. Populations at high altitude in the region, as the people living in Bogotá, Colombia (at an altitude of 2,640 meters [8,660 feet] above sea level), are adapted to hypobaric hypoxia and may have different features of SARS-CoV-2 pneumonia. Identifying regional clinical features of COVID-19 is essential to expand the knowledge to set health policies. We aimed to describe the demographic and clinical characteristics and to explore risk factors of death, intensive care unit (ICU) admission, and use of invasive mechanical ventilation in hospitalized patients diagnosed with SARS-CoV-2 pneumonia in Bogotá, Colombia.

## 2. Materials and Methods

### 2.1. Study Design and Participants

This concurrent cohort study was conducted in a consecutive sample of hospitalized individuals at a single tertiary center that provided care to people with SARS-CoV-2 pneumonia living in Bogotá, Colombia, from March 20, 2020, to June 30, 2020, with a follow-up time of 28 days.

Patients 18 years or older admitted to hospitalization with diagnosis compatible with community-acquired pneumonia and a reverse transcription polymerase chain reaction (RT-PCR) test for SARS-CoV-2 positive in nasopharyngeal swabs were included. Patients with viral coinfection were included, as long as SARS-CoV-2 infection was isolated. We excluded patients that did not have diagnostic imaging to corroborate the diagnosis of pneumonia. Patients transferred to other hospitals were excluded because we were unable to track their outcomes. Patients transferred from other hospitals 48 hours after their initial hospital admission were excluded as well, because we were unable to collect their clinical characteristics and laboratory tests on admission.

### 2.2. Outcomes

The primary outcome was in-hospital death within 28 days of admission. Patients still in hospital at the latest follow-up point on July 28, 2020, were censored for analyses. Once discharged, patients were considered no longer at risk of death. Secondary outcomes included ICU admission and use of invasive mechanical ventilation via an endotracheal or tracheostomy tube.

### 2.3. Data Collection

Patients were included through active detection of results of RT-PCR tests for SARS-CoV-2. Demographic data, clinical characteristics, self-reported underlying comorbidities, laboratory tests on admission (white blood cell count, neutrophil count, lymphocyte count, hemoglobin levels, platelet count, D-dimer levels, lactate dehydrogenase, C-reactive protein, ferritin, procalcitonin, high-sensitivity troponin, liver and kidney function assessment, and arterial blood gas analysis), diagnostic images (chest X-ray and chest CT scan), treatments for viral pneumonia (antiviral therapy, corticosteroids, antibiotics, ventilatory support, vasopressor support, and renal replacement therapy), and outcomes were extracted from electronic medical records. Two researchers independently reviewed the records to double-check the collected data.

Date of illness onset was the first day of symptoms. We used reference values at an altitude of 2,640 meters above sea for assessment of arterial blood gases and hemoglobin levels [5, 6]. Diagnostic image files were analyzed and classified by the investigators. Chest X-ray features were classified as compatible with viral pneumonia (peripheral ground-glass opacities or consolidations, bilateral or unilateral), compatible with an alternative diagnosis (single lobar consolidation, cavitation, nodules, masses, or reticular pattern) or nonspecific (perihilar ground-glass opacities or consolidations or diffuse ground-glass opacities) [7]. Chest CT features were classified as compatible with viral pneumonia (CO-RADS categories 4 and 5), compatible with an alternative diagnosis (CO-RADS categories 1 and 2), or nonspecific (CO-RADS category 3) [8].

### 2.4. Sample Size

The sample size was based on data published on rates of in-hospital mortality (11.7 to 28.3%) and clinical risk factors associated with death in hospitalized patients with SARS-CoV-2 pneumonia [9, 10]. We estimated that it would be necessary to include 327 participants which would provide 80% power, with a 0.05 significance level, to detect an odds ratio of 2.46 of having a higher risk of death at 28 days. The sample size calculation was computed using Epi Info™ version 7.2.3.1 of 2019.

### 2.5. Statistical Analysis

A descriptive analysis of the variables of interest was conducted to report the categorical data by the distribution of frequencies, relative frequencies, and proportions. Continuous variables were expressed as means (standard deviation, SD) and medians (interquartile range, IQR), depending on their distribution. Comparisons between survivors and nonsurvivors groups were tested by *t*-test for continuous variables and by Pearson's chi-squared test or Fisher's exact test for categorical variables.

To evaluate the relationship between variables considered as risk factors and the outcomes binary logistic regression methods were performed. Quantitative variables distributed not normally were categorized for logistic regression, according to cut-off points used in previous studies with COVID-19 populations. After assessment of collinearity and reduction of input variables by a component matrix, twelve factors with the strongest statistical association with the outcomes on bivariate analysis (*p* values <0.05) were included in the multivariate analysis.

All reported *p*-values were two-tailed and calculated with statistical significance set to *p* < 0.05. Statistical analyses were performed using SPSS Statistics version 25.0 (SPSS, Chicago, IL, USA).

### 2.6. Ethical Approval

The study was approved by the local Ethics Committee of Universidad del Rosario (Approved no. DVO005-1230-CV1269), in accordance with the principles of the Declaration of the Helsinki, and the Proposed International Ethical Guidelines for Biomedical Research Involving Human Subjects of the CIOMS/WHO. Written informed consents were taken from the patients' admissions for data collection. The information provided by the patients was confidential.

## 3. Results

Between March 25 and June 30, 2020, 377 adults were admitted with laboratory-confirmed SARS-CoV-2 pneumonia, 214 (56.8%) were male (Table 1). Participants were aged 24–100 years; the mean age was 59.7 years (SD 16.4). At the end of follow-up, six patients were still in the hospital. The first RT-PCR test for SARS-CoV-2 was positive in 368 patients (97.6%), while nine patients (2.4%) had a negative first test and positive second test.

**Figure 1:**
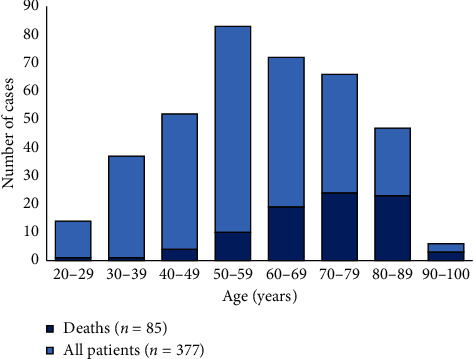
Cases and deaths distribution by age of patients with SARS-CoV-2 pneumonia.

There were 85 (22.6%) deaths. Patients who died were older on average than survivors (*p* ≤ 0.001) (Figure 1). Deaths were more likely in male than female patients (*p*=0.029), but the proportion of women in deaths increased as the population aged.

The median time from first symptom to emergency department admission was 7 days (IQR 4–9). The most common symptoms upon admission included cough, fever, dyspnea, and asthenia (Table 1). Most patients had at least one comorbidity (333 [88.3%]). Arterial hypertension and diabetes mellitus were one of the most common comorbidities. Two out of three patients suffered from overweight (body mass index between 25.0 and 29.9) or obesity (body mass index of 30.0 or higher). Severity of pneumonia evaluated on admission was mild in 251 patients according to CURB-65 score (0 to 1, 73.3%), and 342 had low risk for in-hospital mortality according to a quick SOFA score (0 to 1, 96.3%).

Regarding the most remarkable laboratory findings upon admission to the emergency room, almost half of patients had lymphopenia (lymphocyte count less than 1,000 cells/*μ*L) and it occurred more frequently in nonsurvivors than in survivors (*p* ≤ 0.01) (Table 2). Median concentrations of some systemic inflammation markers were more elevated in nonsurvivors than in survivors, such as lactate dehydrogenase (*p*=0.005), C-reactive protein (*p*=0.017), and procalcitonin (*p*=0.005). Likewise, median D-dimer level was higher in nonsurvivors than in survivors (*p*=0.003).

Chest X-ray was compatible with the suspected diagnosis of pneumonia in 257 (72.6%) patients, and it was normal in 63 (17.8%) patients (Table 2). Chest CT scans were done in 344 patients and CT pulmonary angiographies in 66 patients. Chest CT was compatible with the suspected diagnosis of viral pneumonia in 323 (93.9%) patients. Pulmonary embolism was diagnosed in six cases (1.6%), and all of them were survivors.

Pharmacological treatment of patients admitted to hospital with COVID-19 changed over time during enrollment, according to the suggestions of national clinical guidelines. In this study, 248 (65.8%) patients received systemic corticosteroid therapy, its use was more common in nonsurvivors than in survivors (73 [85.9%] vs. 175 [59.9%]; *p*=0.027). Azithromycin (174 [46.2%]), hydroxychloroquine (85 [22.5%]), and lopinavir/ritonavir (79 [21.0%]) were used less frequently.

Regarding the clinical outcomes, a high proportion of patients (158 [41.9%]) were transferred to the ICU; the median ICU length of stay was 8 (IQR 3–15) days (Table 1). Overall, 123 (32.6%) patients received invasive mechanical ventilation and 116 (30.8%) patients received vasopressor therapy. Renal replacement therapy due to sepsis-associated acute renal failure was necessary in 33 (8.8%) patients. The median length of hospital stay was 9 (IQR 6–15) days.

**Figure 2:**
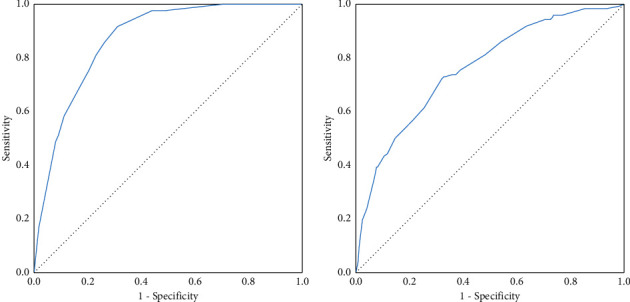
Receiver operating characteristics (ROC) curves for (a) the model of age, ICU admission, and arterial pH for in-hospital mortality (area under the curve 0.869), and (b) the model of age, male sex, peripheral oxygen saturation, and white blood cell count for invasive mechanical ventilation due to SARS-CoV-2 pneumonia (area under the curve 0.761).

After reduction of input variables age, sex, leukocytosis, history of arterial hypertension, COPD or chronic kidney disease, altered mental status on admission, decreased arterial pH, low levels of peripheral oxygen saturation (SpO2), elevated D-dimer levels, nosocomial bacterial infection, and ICU admission had the strongest association with in-hospital death in bivariate analysis. In the multivariable logistic regression analysis, we found that age over 65 years (reference age <50 years, OR 9.26, 95% CI 3.29–26.01; *p* ≤ 0.01) and ICU admission (OR 12.37, 95% CI 6.08–25.18; *p* ≤ 0.01) were associated with increased risk of death; arterial pH higher than 7.47 (reference pH < 7.40, OR 0.25, 95% CI 0.08–0.74; *p*=0.01) on admission was associated with lower risk of death (Table 3). The logistic model of age, arterial pH, and ICU admission had a high discrimination ability for in-hospital death (area under the receiver operating characteristic curve of 0.869) (Figure 2). As a proportion of patients did not have measurements on admission of biomarkers such as procalcitonin and ferritin, a sensitivity analysis including these biomarkers for testing the effect of missing data resulted in similar results.

Age over 65 years, male sex, white blood cell count over 10,000 per *µ*L, and SpO2 lower than 90% on admission were associated with the use of invasive mechanical ventilation (Table 4). The logistic model of age, male sex, SpO2, and white blood cell count had an acceptable discrimination for invasive mechanical ventilation (area under the receiver operating characteristic curve of 0.761) (Figure 2). There were no independent risk factors associated with ICU admission due to COVID-19 in the multivariate analysis.

## 4. Discussion

To our knowledge, this single-center study is the first report of hospitalized adult patients with SARS-CoV-2 pneumonia in Andean subregion in a high-altitude population from Bogotá, Colombia. We observed that COVID-19 hospitalized patients were more likely men over 50 years of age. Demographic characteristics and symptoms of COVID-19 were similar to previous reported data from patients admitted to hospitalization in China, United States, and the UK [11–14]. In our study, in-hospital mortality was 22.6%; age, ICU admission and arterial pH were factors associated with this outcome.

Even though mortality in the present study was consistent with what has been reported, severity of respiratory failure seemed to be worse considering the high proportion of patients admitted to ICU (41.9%) and use of invasive mechanical ventilation (32.6%) in comparison to what was reported in China (26% and 17%, resp.), New York (14.2% and 12.2%, resp.), and the UK (17% and 10%, resp.) [10, 13, 14]. Since the decision of transfer to ICU was based on individual clinical assessment by each physician in general ward or emergency department, we cannot exclude the possibility that some patients with mild disease course had been admitted to ICU; however, the proportion of patients transferred to ICU is correlated with the high proportion of patients who required mechanical ventilation or vasopressor therapy. Severity of respiratory failure could be partially explained because one-third (34.2%) of our patients did not receive corticosteroid therapy for COVID-19, because part of our population was enrolled before the release of the RECOVERY trial report. In the dexamethasone group in the RECOVERY trial the use of invasive mechanical ventilation was way lower (5.7%) than in the present study, but even so mortality was similar (22.9%) [15].

In Latin America, several reports have found a case fatality rate and mechanical ventilation use around 24% in hospitalized patients in Brazil [16, 17]. In the COALITION II trial that assessed efficacy and safety of adding azithromycin to COVID-19 treatment in Brazilian patients, mortality rate and use of mechanical ventilation were even higher to what we showed (40% and 52% in the control group, resp.) [18].

It has been suggested that some local factors in Latin America could influence clinical presentation of COVID-19 in comparison to Europe, such as the younger age of populations, tropical climate, and the immune regulation induced by helminthic infections or extensive BCG vaccination [19, 20]. Colombia has a lower proportion of population over 60 years (13%) in comparison to Italy (29%) or Spain (25%), but at the same time, a lower hospital bed to population ratio and a fragmented healthcare system [21]. These environmental and physiological characteristics may affect the course of COVID-19.

Moreover, observational studies have been suggesting that high altitude is associated with infectivity and case fatality rate of COVID-19, due to factors such as adaptation to chronic hypobaric hypoxia, angiotensin-converting enzyme 2 expression, ultraviolet radiation, and vitamin D production [22]. However, results are conflicting and may be explained by differences in population density, underreporting of cases, and barriers of access to healthcare among populations [23–25]. Although altitude does not affect the mortality rate in general patients undergoing invasive mechanical ventilation, specific features of subgroups of patients with acute respiratory distress syndrome in COVID-19 may influence the need of ventilatory support at high altitude [26]. We theorize that high-altitude hypoxemia could have impacted the severity and course of acute respiratory failure in our study.

On the other hand, this study was conducted in a tertiary care center with one of the largest ICU in Bogotá, so presumably we admitted more severe patients prone to invasive mechanical ventilation from the area. The median duration of symptoms before admission (7 days [IQR 4–9]) was a little bit higher to what was reported in New York and the UK [13, 14]; factors not yet assessed and involved in late admission of COVID-19 patients could have affected our results.

In our study, most patients had a mild pneumonia on admission, according to CURB-65 and qSOFA scores. Zhou et al. [10] described in a cohort of 191 patients in Wuhan a CURB-65 score of 0 to 1 in most of them (75%) as well. It is possible that clinical prediction rules traditionally used to evaluate severity of community-acquired pneumonia may underestimate risk of mortality or ICU admission in SARS-CoV-2 pneumonia, since they were not developed to predict outcomes in viral pneumonia. Clinical deterioration in COVID-19 occurs later in comparison to bacterial pneumonia (in the present study, 9 days from illness onset to ICU admission), so prediction rules at admission might be inaccurate. Data published is conflicting about the performance of these prediction rules in COVID-19 [27–30]. Scores developed for viral pneumonia, such as MuLBSTA, 4C, or CALL, may better predict the severity in this subset of patients, although they have not been validated in high-altitude populations [30–33].

In the logistic models developed in this study, age and male sex were associated with COVID-19 severity; these results are consistent with risk factors for poor prognosis previously reported [14, 34]. Inflammatory biomarkers, such as C-reactive protein, ferritin, and procalcitonin, have been associated with mortality among COVID-19 patients [34, 35]. Likely, since some of our patients did not have these markers measured on admission, we could not validate them as independent risk factors. On the other hand, biological variations on biomarkers due to different ethnic backgrounds might modify their prognostic ability in populations like ours. Regarding laboratory findings, in our model for mortality pH in arterial blood gas test on admission was validated as an important biomarker, and this factor had not been associated with severe disease before. Sepsis-induced tissue hypoperfusion or diaphragmatic fatigue leading to hypercapnia might be the cause acidosis on admission in patients with severe COVID-19. Most studies in COVID-19 like this have assessed prognostic markers on admission, and further studies should address the diagnostic accuracy of markers follow-up.

There are some limitations to our study. First, clinical data collected relied on medical records which might lead to misclassification or recall biases. Nevertheless, we verified thoroughly the collected data; significant underreporting was unlikely because report of clinical characteristics and underlying comorbidities was consistent with existing literature. Second, there were missing data of symptoms and laboratory findings in some cases. This limitation is common in observational studies and might contribute to the underestimation of the true strength of any association. Third, the power of statistical analyses may have been affected by the sample size and categorization of variables. Fourth, this study was conducted with hospitalized patients in a single tertiary care center in a high-altitude city, so it is possible that the sickest patients with highest degree hypoxemia were admitted. Patients were included by convenience sampling during the first months of the pandemic to describe the characteristics in our center; thus, our population is not representative of the general population through the whole pandemic. Caution should be exercised about generalizing these data to different settings. Finally, due to the study design we cannot establish a causal effect between risk factors and outcomes; our results and the model developed need a prospective validation.

## 5. Conclusions

In this single-center study of hospitalized patients living at high altitude with SARS-CoV-2 pneumonia, clinical characteristics were consistent with existing data. Mortality was similar to what has been reported; however, ICU admission and use of invasive mechanical ventilation were higher.

Factors associated with in-hospital death as increasing age, arterial pH, and ICU admission could help to identify patients with poor prognosis. Further studies may help to understand the usefulness of biomarkers follow-up in prognosis and the impact of high altitude in severity of COVID-19.

## Figures and Tables

**Table 1:** Characteristics on admission to hospital and outcomes of the study population.

	Total (*n* = 377)	Survivor (*n* = 292)	Nonsurvivor (*n* = 85)
*Age, years*	59.7 ± 16.4	56.7 ± 15.8	70.1 ± 13.9
<50	103 (27.3)	97 (33.2)	6 (7.1)
50–65	130 (34.5)	108 (37.0)	22 (25.9)
>65	144 (38.2)	87 (29.8)	57 (67.1)

*Sex*			
Female	163 (43.2)	135 (46.2)	28 (32.9)
Male	214 (56.8)	157 (53.8)	57 (67.1)

*Symptoms*			
Cough	335/360 (93.1)	262/281 (93.2)	73/79 (92.4)
Fever	280/346 (80.9)	219/272 (80.5)	61/74 (82.4)
Dyspnea	258/294 (87.8)	191/222 (86.0)	67/72 (93.1)
Asthenia	136/137 (99.3)	117/118 (99.2)	19/19 (100.0)
Sore throat	106/153 (69.3)	91/128 (71.1)	15/25 (60.0)
Diarrhea	77/154 (50.0)	63/121 (52.1)	14/33 (42.4)

*Comorbidities*			
Arterial hypertension	149 (39.5)	99 (33.9)	50 (58.8)
Overweight	145 (38.5)	122 (58.2)	23 (27.1)
Obesity	114 (30.2)	90 (30.8)	24 (28.2)
Diabetes mellitus	82 (21.8)	55 (18.8)	27 (31.8)
Chronic obstructive pulmonary disease	34 (9.0)	17 (5.8)	17 (20.0)
Chronic kidney disease	19 (5.0)	10 (3.4)	9 (10.6)
Coronary artery disease	18 (4.8)	13 (4.5)	5 (5.9)
Heart failure	17 (4.5)	10 (3.4)	7 (8.2)

*Number of comorbidities*			
0	44 (11.7)	32 (11.0)	12 (14.1)
1	169 (44.8)	130 (44.5)	39 (45.9)
2	83 (22.0)	63 (21.6)	20 (23.5)
3	57 (15.1)	48 (16.4)	9 (10.6)
≥4	24 (6.4)	19 (6.5)	5 (5.9)

*Smoking status*			
Current smoking	7 (1.9)	6 (2.1)	1 (1.2)
Former smoking	38 (10.1)	28 (9.6)	10 (11.8)
Body mass index	27.3 (24.1–30.9)	27.4 (24.8–31.0)	25.8 (22.8–30.9)

*Baseline vital signs*			
Systolic blood pressure, mmHg	128.0 (116.0–140.0)	127.0 (115.0–138.0)	130.0 (119.3–151.8)
Heart rate, bpm	96.3 ± 17.5	96.0 ± 16.5	97.1 ± 20.7
Respiratory rate, bpm	20 (18–22)	20 (18–21)	20 (19–24)

*Pneumonia severity*			

*CURB-65*			
0 or 1	251/342 (73.4)	215/262 (82.1)	36/80 (45.0)
2	79/342 (23.1)	41/262 (15.6)	38/80 (47.5)
≥3	12/342 (3.5)	6/262 (2.3)	6/80 (7.5)

*qSOFA*			
0 or 1	324/337 (96.3)	267/275 (97.1)	75/80 (93.8)
≥2	13/337 (3.7)	8/275 (2.9)	5/80 (6.2)
Days from illness onset to hospital admission	7 (4–9)	7 (4–10)	6 (4–8)
Days from illness onset to ICU admission	9 (6–11)	9 (7–12)	8 (6–10)

*Outcomes*			
Invasive mechanical ventilation	123 (32.6)	50 (17.1)	73 (85.9)
ICU admission	158 (41.9)	85 (29.1)	73 (85.9)
ICU length of stay, days	8 (3–15)	7 (3–14)	10 (3–17)
Length of hospital stay, days	9 (6–15)	9 (6–14)	10 (5–18)

Data are presented as mean ± standard deviation, median (interquartile range), or n/N (%), where N is the total number of patients with available data.

**Table 2:** Laboratory and radiographic findings of the study population on admission to hospital.

	Total (*n* = 377)	Survivor (*n* = 292)	Nonsurvivor (*n* = 85)
*Laboratory findings*			
White blood cell count, ×109/L	7.545 (5.510–10.035)	7.140 (5.380–9.540)	8.550 (6.235–12.075)
<4	24/376 (6.4)	21/291 (7.2)	3/85 (3.5)
4 to 10	257/376 (68.4)	205/291 (70.4)	52/85 (61.2)
>10	95/376 (25.3)	65/291 (22.3)	30/85 (35.3)
Lymphocyte count, ×109/L	1.105 (0.803–1.468)	1.150 (0.890–1.520)	0.810 (0.560–1.250)
<1	156/376 (41.5)	102/291 (35.1)	54/85 (63.5)
≥1	220/376 (58.5)	189/291 (64.9)	31/85 (36.5)
Hemoglobin, mg/dL	14.8 ± 2.0	14.9 ± 1.8	14.5 ± 2.6
<12.5	38/376 (10.1)	20/291 (6.9)	18/85 (21.2)
≥12.5	338/376 (89.9)	271/291 (93.1)	67/85 (78.8)
Platelet count, ×109/L	218.000 (177.000–274.750)	225.000 (179.000–281.000)	196.000 (159.000–251.500)
<100	9/376 (2.4)	7/291 (2.4)	2/85 (2.4)
≥100	367/376 (97.6)	284/291 (97.6)	83/85 (97.6)
Lactate dehydrogenase, U/L	344.0 (266.5–462.0)	325.0 (258.0–414.5)	458.0 (310.0–646.0)
≤250	70/365 (19.2)	63/282 (22.3)	7/83 (8.4)
>250	295/365 (80.8)	219/282 (77.7)	76/83 (91.6)
Ferritin, ng/mL	854.0 (472.0–1500.0)	768.5 (470.8–1406.5)	1131.0 (512.5–1942.5)
<600	112/315 (35.6)	90/238 (37.8)	22/77 (28.6)
≥600	203/315 (64.4)	148/238 (62.2)	55/77 (71.4)
C-reactive protein, mg/L	112.3 (63.6–181.7)	101.3 (55.1–167.2)	143.8 (99.1–226.1)
<50	58/309 (18.8)	52/241 (21.6)	6/68 (8.8)
≥50	251/309 (81.2)	189/241 (78.4)	62/68 (91.2)
Procalcitonin, ng/mL	0.27 (0.11–0.93)	0.18 (0.09–0.59)	0.73 (0.25–1.84)
<0.1	15/70 (21.4)	14/48 (29.2)	1/22 (4.5)
0.1 to <0.5	28/70 (40.0)	20/48 (41.6)	8/22 (36.4)
≥0.5	27/70 (38.6)	14/48 (29.2)	13/22 (59.1)
D-dimer, mg/L	0.49 (0.28–0.93)	0.46 (0.27–0.90)	0.65 (0.37–1.14)
<0.5	184/364 (50.6)	156/283 (55.1)	28/81 (34.6)
0.5 to 1.0	101/364 (27.7)	71/283 (25.1)	30/81 (37.0)
>1.0	79/364 (21.7)	56/283 (19.8)	23/81 (28.4)
Creatinine, mg/dL	0.97 (0.79–1.21)	0.90 (0.76–1.12)	1.19 (0.96–1.58)
<1.5	314/363 (86.5)	254/278 (91.4)	60/85 (70.6)
≥1.5	49/363 (13.5)	24/278 (8.6)	25/85 (29.4)
Alanine aminotransferase, U/L	35.0 (22.0–58.5)	38.0 (22.8–61.0)	30.5 (18.3–51.8)
≤40	181/314 (57.6)	128/234 (54.7)	53/80 (66.3)
>40	133/314 (42.4)	106/234 (45.3)	27/80 (33.7)
Arterial pH	7.44 (7.42–7.46)	7.44 (7.42–7.47)	7.43 (7.4–7.46)
<7.40	37/370 (10.0)	19/286 (6.6)	18/84 (21.4)
7.40–7.47	277/370 (74.9)	221/286 (77.3)	56/84 (66.7)
>7.47	56/370 (15.1)	46/286 (16.1)	10/84 (11.9)
SpO2, %	90.5 (86.9–93.9)	91.0 (87.0–94.0)	90.0 (84.2–92.8)
<90	161/370 (38.4)	121/286 (42.3)	40/84 (47.6)
≥90	209/370 (61.6)	165/286 (57.7)	44/84 (52.4)
PaO2/FiO2 ratio	239.0 (198.0–270.0)	246.5 (213.8–274.0)	188.5 (152.5–239.8)
<100	18/370 (4.9)	5/286 (1.7)	13/84 (15.5)
100 to 199	77/370 (20.8)	44/286 (15.4)	33/84 (39.3)
200 to 299	236/370 (63.8)	204/286 (71.3)	32/84 (38.1)
≥300	39/370 (10.5)	33/286 (11.5)	6/84 (7.1)
Viral coinfection	4/63 (6.3)	3/34 (8.8)	1/29 (3.4)
Bacterial coinfection	10/109 (9.2)	5/65 (7.7)	5/44 (11.4)

*Chest X-ray features*			
Normal	63/354 (17.8)	56/271 (20.7)	7/83 (8.4)
Compatible with pneumonia	257/354 (72.6)	187/271 (69.0)	70/83 (84.3)
Compatible with an alternative diagnosis	10/354 (2.8)	8/271 (2.9)	2/83 (2.4)
Nonspecific features	24/354 (6.8)	20/271 (7.4)	4/83 (4.8)

*Chest CT features*			
Compatible with viral pneumonia	323/344 (93.9)	261/274 (95.3)	62/70 (88.6)
Compatible with an alternative diagnosis	15/344 (4.4)	11/274 (4.0)	4/70 (5.7)
Nonspecific features	6/344 (1.7)	2/274 (0.7)	4/70 (5.7)
Pulmonary embolism on CT pulmonary angiography	6/66 (1.6)	6/57 (2.1)	0/9 (0.0)

Data are presented as mean ± standard deviation, median (interquartile range), or n/N (%), where N is the total number of patients with available data.

**Table 3:** Multivariate analysis of risk factors associated with in-hospital death in patients with SARS-CoV-2 pneumonia.

Factors	OR (95% CI)	*p* value
*Age, years*		
<50	1 (ref)	≤0.001
50–65	2.64 (0.89–7.87)	0.08
>65	9.26 (3.29–26.01)	≤0.001

*Arterial pH*		
<7.40	1 (ref)	0.03
7.40–7.47	0.43 (0.19–0.97)	0.04
>7.47	0.25 (0.08–0.74)	0.01

*ICU admission*	12.37 (6.08–25.18)	≤0.001

**Table 4:** Multivariate analysis of risk factors associated with invasive mechanical ventilation in patients with SARS-CoV-2 pneumonia.

Factors	OR (95% CI)	*p* value
*Age, years*		
<50	1 (ref)	≤0.001
50–65	2.05 (1.03–4.10)	0.04
>65	5.25 (2.66–10.36)	≤0.001
*Male sex*	2.36 (1.40–3.97)	0.001

*SpO_2_ <90%*	0.38 (0.23–0.63)	≤0.001

*White blood cell count, ×10* ^*9*^ */L*		
<4	1 (ref)	≤0.001
4 to 10	1.77 (0.47–6.64)	0.40
>10	5.73 (1.46–22.46)	0.01

## Data Availability

All the data used to support the findings of this study are available from the corresponding author on reasonable request.
